# Volatile β-Ocimene Can Regulate Developmental Performance of Peach Aphid *Myzus persicae* Through Activation of Defense Responses in Chinese Cabbage *Brassica pekinensis*

**DOI:** 10.3389/fpls.2018.00708

**Published:** 2018-05-28

**Authors:** Zhi-Wei Kang, Fang-Hua Liu, Zhan-Feng Zhang, Hong-Gang Tian, Tong-Xian Liu

**Affiliations:** ^1^State Key Laboratory of Crop Stress Biology for Arid Areas, Key Laboratory of Northwest Loess Plateau Crop Pest Management of Ministry of Agriculture, Northwest A&F University, Xianyang, China; ^2^State Key Laboratory of Integrated Management of Pest Insects and Rodents, Institute of Zoology, Chinese Academy of Sciences, Beijing, China

**Keywords:** β-ocimene, Chinese cabbage, *Myzus persicae*, phytochemical, *Aphidius gifuensis*

## Abstract

In nature, plants have evolved sophisticated defense mechanisms against the attack of pathogens and insect herbivores. Plant volatile-mediated plant-to-plant communication has been assessed in multitrophic systems in different plant species and different pest species. β-ocimene is recognized as an herbivore-induced plant volatile that play an important role in the chemical communication between plants and pests. However, it is still unclear whether β-ocimene can active the defense mechanism of Chinese cabbage *Brassica pekinensis* against the peach aphid *Myzus persicae*. In this study, we found that treatment of Chinese cabbage with β-ocimene inhibited the growth of *M. persicae* in terms of weight gain and reproduction. Moreover, β-ocimene treatment negatively influenced the feeding behavior of *M. persicae* by shortening the total feeding period and phloem ingestion and increasing the frequency of stylet puncture. When given a choice, winged aphids preferred to settle on healthy Chinese cabbage compared with β-ocimene-treated plants. In addition, performance of the parasitoid *Aphidius gifuensis* in terms of Y-tube olfaction and landings was better on β-ocimene-treated Chinese cabbage than on healthy plants. Furthermore, β-ocimene significantly increased the expression levels of salicylic acid and jasmonic acid marker genes and the accumulation of glucosinolates. Surprisingly, the transcriptional levels of detoxifying enzymes (CYP6CY3, CYP4, and GST) in aphids reared on β-ocimene-treated Chinese cabbage were significantly higher than those of aphids reared on healthy plants. In summary, our results indicated that β-ocimene can activate the defense response of Chinese cabbage against *M. persicae*, and that *M. persicae* can also adjust its detoxifying enzymes machinery to counter the host plant defense reaction.

## Introduction

In nature, plant volatiles play a pivotal role in the interactions of plants and environment, such as the attraction of pollinators and seed dispersers and protection against insect herbivores and pathogens (Heil and Karban, 2010; Holopainen and Blande, 2013; Loreto et al., 2014; Pierik et al., 2014). Generally, vegetables release certain volatiles in response to attack by insect herbivores and pathogens (Heil and Karban, 2010; Holopainen and Blande, 2013; Pierik et al., 2014). These induced volatiles can attract the natural enemies of insect herbivores, repel other insect herbivores and increase the resistance of tissues in both the host plant and neighboring plants (Heil and Silva Bueno, 2007; Heil and Karban, 2010; Pieterse et al., 2013). For example, the parasitoid *Aphidius gifuensis* has been demonstrated to show a preference for wheat infested with its host *Sitobion avenae* compared with healthy wheat (Pan and Liu, 2014). Furthermore, *Cardiochiles nigriceps*, which is a parasitic wasp of *Heliothis virescens*, can distinguish between *H. virescens*- and *Helicoverpa zea*-induced plant volatiles to locate its host directly (De Moraes et al., 1998). In addition, several volatiles released from *H. virescens*-damaged tobacco plants *Nicotiana tabacum* at night are highly repellent to the adult females of this moths (De Moraes et al., 2001). Furthermore, intermittent exposure to the volatiles emitted from artificially damaged *Arabidopsis* has been shown to induce defensive responses in undamaged neighboring plants (Shiojiri et al., 2012).

Generally, herbivore-induced volatile blends are dominated by terpenes (or terpenoids) (Hare, 2011; Mithofer and Boland, 2012; Holopainen and Blande, 2013). A diverse array of terpenes emitted in response to insect herbivore attack have been detected in various plants and insect herbivore species (De Moraes et al., 1998; Mithofer and Boland, 2012; Ponzio et al., 2014). For example, the feeding of *Pieris brassicae* significantly increased the emission of (E,E)-TMTT (4,8,12-trimethyl-1,3,7,11-tridecatetraene) and α-barbatene whereas the feeding of *Brevicoryne brassicae* decreased the release of these volatiles (Ponzio et al., 2014). Furthermore, *N. tabacum* damaged by *H. zea* released more (E,E)-TMTT and (E,E)-α-farnesene than plants damaged by *H. virescens*, whereas *N. tabacum* damaged by *H. virescens* released more α-pinene than that damaged by *H. zea* (De Moraes et al., 1998). Among these terpenes, β-ocimene is one of the most well documented and important plant volatiles induced in response to herbivory (Arimura et al., 2000; De Moraes et al., 2001; Ponzio et al., 2014; Takemoto and Takabayashi, 2015). For example, pre-exposure to (Z)-jasmone significantly triggered the emission of β-ocimene in broad bean and resulted in a higher attractive of female *Aphidius ervi* (Birkett et al., 2000). A wind tunnel bioassay subsequently confirmed that female *A. ervi* show a higher preference for pure β-ocimene (Birkett et al., 2000). In addition, adult female *Gonatocerus ashmeadi*, which is an egg parasitoid of *Homalodisca vitripennis* (Germar), have been shown to be highly attracted to a mixture of E-β-ocimene and (E,E)-α-farnesene (Krugner et al., 2014). Collectively, these previous studies have indicated that β-ocimene is neutral with respect to the chemical preferences of natural enemies, including parasitoids and predators (Krugner et al., 2014; Takemoto and Takabayashi, 2015).

Exposure to β-ocimene has also been found to trigger plant defense responses via the signaling pathways of salicylic acid (SA), jasmonic acid (JA), and ethylene (ET), which together constitute the hormonals backbone of plant defense responses that influence plant–insect interactions (Kishimoto et al., 2005). For example, when undamaged tomatoes were exposed to (E)-β-ocimene emitted from transgenic tobacco plants, they are found to be more attractive to *A. ervi* than are those plants exposed to normal tobacco (Cascone et al., 2015). Similarly, exposure to β-ocimene significantly increased the expression of *Arabidopsis* defense genes [e.g., pathogenesis-related protein (*PR*), allene oxide synthase (*AOS*), lipoxygenase (*LOX*), and vegetative storage protein (*VSP*)] and downstream metabolism (camalexin) to increase the resistance against *Botrytis cinerea* (Kishimoto et al., 2005, 2006). Furthermore, in *Apis mellifera*, β-ocimene, also acts as a pheromone in young larvae and modify the behavioral maturation of workers (Maisonnasse et al., 2010). β-ocimene has also been shown to increase both mating and oviposition rates in *Hyphantria cunea* (Tang et al., 2016). In addition, preference tests using E-β-ocimene or (E,E)-α-farnesene alone have indicated that a 1:1 ratio of these volatiles enhanced the first choice, number of visits, and residence time of parasitoids compared with each volatile alone or the control (Krugner et al., 2014). All of these results indicated that plants might be able to utilize airborne information to adjust their defensive arsenal to fend off attackers.

To cope with plant defense responses, insect pests have in turn been found to adjust their feeding behavior and deploy certain detoxification systems, including detoxifying enzymes and xenobiotic transporters to counter the plant allelochemicals (or toxic compounds) (Francis et al., 2005; Schuler and Berenbaum, 2013; Hassan et al., 2015). For example, in *Spodoptera litura*, feeding with plant allelochemicals induced the gene expressions of Cytochrome P-450 families: *CYP6B48*, *CYP6B58*, *CYP6AB14*, and *CYP9A40*, and RNAi experiments have also revealed that knock down of *CYP6AB14* significantly reduced the resistance of *S. litura* to these plant alleochemicals (Wang et al., 2015a,b,c). Similarly, *CYP6DA2* in *Aphis gossypii* has been identified to be involved in tolerance to gossypol (Peng et al., 2016b). Furthermore, in *Myzus persicae*, *CYP6CY3* has been identified to participate in the detoxification of plant allelochemicals and certain synthetic insecticides (Bass et al., 2013; Peng et al., 2016a). In addition to these cytochrome P450 enzymes, other detoxification enzymes, such as glutathione *S*-transferases (GSTs) also play very important roles in this process. For example, *GSTE1* in *S. litura* catalyzes the plant secondary metabolites: indole-3-carbinol and allyl-isothiocyanate *in vitro* (Xu et al., 2015). Moreover, knocked down of *GSTE1* negatively influenced the feeding behavior and larval weight of *S. litura* feeding on different plants (Xu et al., 2015). In addition, in *A. mellifera*, exposure of nicotine significantly induced the expression of heat-shock proteins (HSPs), including *HSP 70* and *HSP 90* (Esther et al., 2015). Collectively, these results revealed that insect pests have evolved specific counter defenses in response to the defensive chemistry of plants.

Chinese cabbage, *Brassica pekinensis* is one of the most commonly grown and important commercial vegetables in Asia, particularly in East Asia. *M. persicae* is among the most destructive pests of *B. pekinensis* (Van Emden and Harrington, 2017). As the main pest control, the extensive application of chemical insecticides has resulted in the rapid development of insecticide resistance in this aphid pest (Silva et al., 2012). Given this scenario, plant volatile-based insect pest control is considered to be an alternative method of crop protection. In this study, we examined the interaction between Chinese cabbage and *M. persicae* to evaluate potential application of the plant volatile β-ocimene. We initially assessed the activation effect of β-ocimene on plant immune response and then analyzed the influence of β-ocimene on the orientation behavior of winged aphid and a parasitic wasp, *Aphidius gifuensis*, which is an endoparasitoid of *M. persicae* and has been used as a biological agent in the field and greenhouse (Yang et al., 2011; Kang et al., 2017a). Furthermore, we also investigated how *M. persicae* responds to the defense responses induced in its host plant. The results of this study will not only help us to gain a better understanding of how plant volatiles influence the crop community but also provide new insights into the potential application forms of key volatile components such as β-ocimene in integrated pest management systems.

## Materials and Methods

### Plant and Insects

As a host plant, we used Chinese cabbage, *Brassica pekinensis*, c.v. ‘QingZa 3’ (Qingdao International Seed Co., Ltd., Qingdao, Shandong, China). The germinated seeds were sown in 250-mL pots placed in a walk-in growth chamber (24 ± 1°C, 60 ± 5% RH and 16L:8D h). Fourteen-day-old plants were used for all experiments.

*Myzus persicae* were reared on Chinese cabbage in a climate-controlled room (24 ± 1°C, 60 ± 5% RH and 16L:8D h).

Individuals of the aphid parasitic wasp *Aphidius gifuensis* Walker were collected from mummified *M. persicae* on Chinese cabbage in a greenhouse in Yangling, Shaanxi, China. All mummified aphids were cultured in culture dishes in the same climate-controlled room of *M. persicae* until wasp emergence. Approximately 50 paired adult wasps were released in a cage (40 cm × 40 cm × 40 cm) containing Chinese cabbages infested with about 500 mixed-age *M. persicae*. All the females used in the Y-tube olfactometer tests and wind tunnel bioassays were between 3 and 5 days old.

### Plant Treatments

Ten microliters of 0.1 M β-ocimene (Sigma-Aldrich Co.) was applied onto a cotton swab, which was then hung in a glass jar (3-L) containing a Chinese cabbage plant. Given that the β-ocimene, we used dissolved in CH_2_Cl_2_, we used CH_2_Cl_2_ treated plants as controls. Plant treated with water only were used as negative controls. Twenty-four hours later, these treated plants were used for the investigation.

### Assessment of Aphid Performance

To assess the influence of β-ocimene treatment on the performance of *M. persicae*, five apterous adult aphids were introduced onto the first leaf of β-ocimene-treated and control plants, respectively (each treatment comprised eight plants). Twenty-four hours later, only five nymphs were left on each leaf and maintained until reaching the adult stage. To prevent the escape of *M. persicae*, the infested leaves were enclosed within cotton bags. Newly emerged adults were weighed on a microbalance (Sartorius, Göttingen, Germany). Each plant was considered a biological replicate and data were obtained from eight plants per treatment. Thereafter, one newly emerged adult was placed back on each leaf and the number of nymphs produced by each adult within 7 days was recorded. In total, we assessed 12 biological replicates in this work.

### Feeding Behavior of *M. persicae*

Given that we observed no difference in the performance of *M. persicae* placed on water- and CH_2_Cl_2_-treated plants, we only compared the feeding behavior of aphids on β-ocimene treated and CH_2_Cl_2_-treated plants, the latter of which were regarded as controls. Feeding behaviors of *M. persicae* were monitored using the electrical penetration graphs (EPG) technique (Wageningen, Netherlands). A pre-starved apterous adult *M. persicae* (60 min) was placed in the central part of a leaf, and the aphid-plant system was placed in a Faraday cage. Each aphid was monitored continuously for 8 h and at least 17 to 18 replicates were successfully assessed for each treatment. Acquisition and analysis of data and the stylet waveform were analyzed using Stylet+ as described by Tjallingii (1978). All behavioral variables were processed using MS Excel Workbook based EPG data analysis software, which was developed by Sarria et al. (2009).

### Olfactometer Bioassays

The responses of *A. gifuensis* females to β-ocimene treated and control plants were assessed through the two choice tests in a Y-tube and wind tunnel, respectively, whereas the responses of winged aphids were only analyzed using a wind tunnel.

In the Y-tube choice assays, β-ocimene treated and control plants were placed in two 3-L glass containers, which were connected to a Y-tube and air generator through odorless tubes. Prior to use, all glass containers and Y-tube olfactometers were rinsed with 95% ethanol and distilled water and dried in a hot oven (60^o^C). Activated charcoal-filtered air was blown into the container at a rate of 200 mL min^-1^, which was controlled by two airflow meters. Female parasitoids were tested individually. Only the parasitoids that traversed two-thirds of the arm of the Y-tube olfactometer within 5 min were recorded as making a successful selection, otherwise they were scored as “no choice.” A total of 100 parasitoids were tested. In order to avoid possible contamination, we replaced all glass vessels and Y-tubes with clean ones and changed the position of the container after batches of 20 individuals had been tested. For the β-ocimene attraction test, a sterile cotton swab soaked with 10 μL β-ocimene or CH_2_Cl_2_ was placed in 3-L glass container and all the experiments were conducted as described above.

The responses of adult winged aphid and female *A. gifuensis* to β-ocimene-treated and control plants were also examined in a wind tunnel as described previously (Islam et al., 2017). In this test, four pots of β-ocimene-treated and control plants were placed in the upwind area of a wind tunnel. For the winged aphids, 40 winged adult *M. persicae* were released at a distance of 0.5 m from the treated plants. All evaluations were performed starting at 10:00 am, and 24 h later, the number of *M. persicae* was carefully counted to compare their preference between β-ocimene treated and control plants in the presence of both visual and olfactory cues. Each experiment was considered a replicate and data were obtained from five replicates. In tests using female *A. gifuensis*, each female was released individually and used only once. The first choice of each female within 5 min was recorded, after which the wasp was removed from the wind tunnel. In total, we assessed the response of 200 female *A. gifuensis* in this work.

### RNA Extraction and Quantitative RT-PCR

TRIzol reagent (Takara Bio, Tokyo, Japan) was used to extract RNA from the first leaves and five adult *M. persicae* and the first strand complementary DNA (cDNA) was synthesized using a PrimeScript^®^ RT reagent Kit with gDNA Eraser (perfect Real Time) (Takara, Tokyo, Japan) and 1 μg of high quality RNA. qRT-PCR was performed using an iQ5 real-time PCR system (Bio-Rad) following a standard protocol with three biological and technical replicates. The primers used in this work were referenced from previous studies (Silva et al., 2012; Cao et al., 2016; Kang et al., 2017b).

### The Effects of β-Ocimene on Expression Levels of JA and SA Marker Genes

Phytohormones such as SA and JA play key roles in plant responses to biotic stress and hormone-responsive genes are used to qualitatively and quantitatively evaluate defense responses during aphid feeding (Mai et al., 2014; Cao et al., 2016). For the purposes of defense-response monitoring, we need to assess the expression of defense-related phytohormone marker genes (Cao et al., 2016). In Chinese cabbage, the expression of *BrLOX2* (the Genbank accession number: EX100417) and *BrVSP2* (the Genbank accession number: EX103556) were triggered by methyl jasmonate (MEJA) and have been identified as JA pathway marker genes, whereas *BrBGL2* (β-1, 3-Glucanase 2; the Genbank accession number: BBRAF10P08) and *BrPR1* (the Genbank accession number: BBRAF03K11) were elicited by SA and have been identified as SA pathway maker genes (Abe et al., 2011; Cao et al., 2016). We accordingly evaluated the expression of these genes. The Actin 2 (*BrACT2*; the Genbank accession number: BBRAF03F20) gene was used as the internal reference gene for normalization (Cao et al., 2016). The first leaves of plants used in each treatment were harvested at 12 and 24 h after treatment (five replicates for each treatment) for qRT-PCR.

### The Effects of β-Ocimene on the Accumulation of Glucosinolates

As the most important defense allelochemicals in crucifer crops, the accumulation of glucosinolates in response to β-ocimene exposure was also evaluated. The leaf tissues used for glucosinolate analysis were collected as described above, and the extraction and analysis of glucosinolates were conducted as described previously (Cao et al., 2016).

### Detoxify Response in *M. persicae*

To assess how *M. persicae* individuals cope with the defense responses in Chinese cabbage, we analyzed the expression of three detoxification enzyme genes: *CYP6CY3*, *CYP4*, and *GST*, and two stress response genes: *HSP70* and *HSP60*. Newly emerged adult aphids were maintained on β-ocimene treated and control plants for 12 h, and then ten *M. persicae* reared on the same plant from each treatment were collected as a single replicate for qRT-PCR. Each treatment was performed with three biological replicates.

### Statistical Analysis

Adult aphid weights, the numbers of nymphs produced by an adult within 7 days, glucosinolate concentration, wind tunnel data for β-ocimene-treated and control plants, and *M. persicae* gene expression were analyzed using either Student’s *t*-test or the non-parametric Mann–Whitney *U* test (*P* < 0.05). A Chi-square test was used to compare differences in Y-tube olfactometer choice bioassays at *P* < 0.05. The expression of genes associated with Chinese cabbage JA and SA signaling pathways and data for settled aphids were analyzed through one-way analysis of variance (ANOVA), with the test of normality and homoscedasticity of data at sig > 0.05 and means being separated using the Tukey test. All analyses were conducted using IBM SPSS version 19.0 (SPSS Inc., Chicago, IL, United States), and all data are presented as the means ± standard errors.

## Results

### β-Ocimene Inhibited the Developmental Performance of Aphid

**FIGURE 1:**
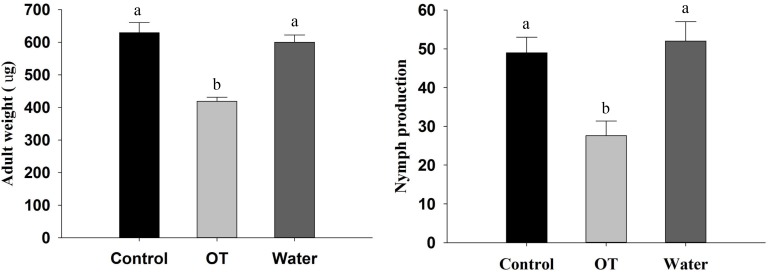
Performance of *M. persicae* reared on β-ocimene treated and control Chinese cabbages. OT, β-ocimene treated Chinese cabbage; CK, CH_2_Cl_2_ treated Chinese cabbage; Water, water treated Chinese cabbage, which was used as negative control. Different letters over the bars designate a significant difference at *P* < 0.05.

To assess the influence of β-ocimene treatment on the performance of *M. persicae*, we analyzed adult weight and nymph production of adult *M. persicae*. The weight of adults on control plants was found to be significantly heavier than that of adults on β-ocimene-treated plants (*F* = 172.025, *P* < 0.001; **Figure 1**). Moreover, the aphids feeding on the control plants produced more offspring compared with those feeding on β-ocimene-treated plants (*F* = 9.406, *P* = 0.014; **Figure 1**). There were, however, no differences in adult weight and the number of nymphs produced by adults feeding on water and CH_2_Cl_2_-treated plants (**Figure 1**).

### β-Ocimene Impaired the Feeding Behavior of Aphid

**FIGURE 2:**
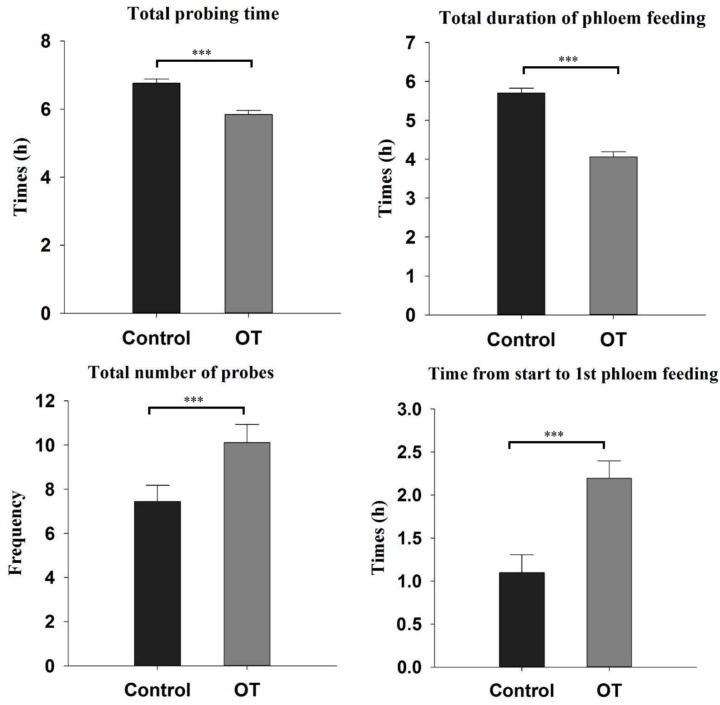
Feeding behavior parameters of *M. persicae* on β-ocimene treated and control Chinese cabbages revealed by electrical penetration graph (EPG). OT, β-ocimene treated Chinese cabbage. Different letters over the bars designate a significant difference: ^∗^*P* < 0.05, ^∗∗^*P* < 0.01, ^∗∗∗^*P* < 0.001.

As β-ocimene inhibited the developmental performance of aphid, we used EPG technology to analyze its impact on the feeding behavior of aphids. EPG analysis showed that *M. persicae* exhibited significantly different feeding behaviors on control and β-ocimene-treated plants (**Figure 2**). The total duration of probing time and phloem feeding of aphids feeding on β-ocimene-treated plants were significantly shorter than those of aphid feeding on control plants (total probing time: *t* = 5.685, *P* < 0.001; total duration of phloem feeding: *t* = 7.641, *P* < 0.001). In contrast, the time from start to 1st phloem feeding of aphids feeding on β-ocimene-treated plants was significantly longer than that in aphids feeding on control plants (*t* = -5.705, *P* < 0.001). In addition, the total number of probes of aphids feeding on β-ocimene-treated plants was significantly greater than that of aphids feeding on control plants (*t* = -2.405, *P* = 0.022).

### β-Ocimene Increased the Attractiveness of Plants to *A. gifuensis* and Repellency to Winged Aphid

**FIGURE 3:**
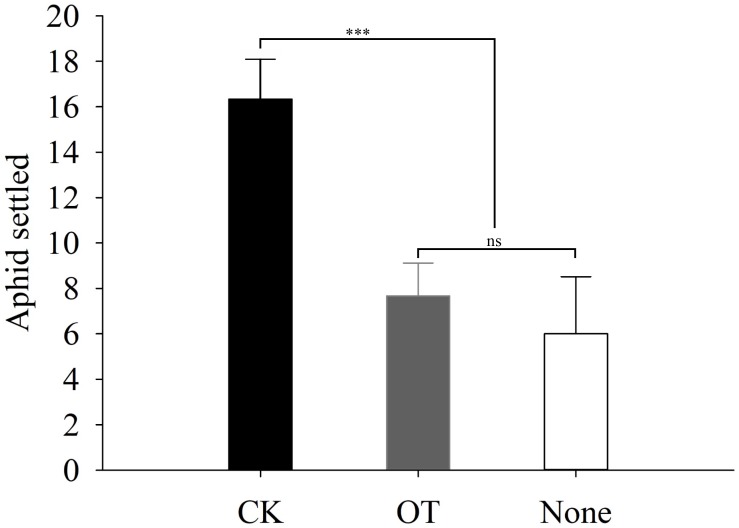
Flight behavior of the winged aphid on β-ocimene-treated and control Chinese cabbages. O, purified β-ocimene. OT, β-ocimene treated Chinese cabbage. None, no choice. Different letters over the bars designate a significant difference: ^∗^*P* < 0.05, ^∗∗^*P* < 0.01, ^∗∗∗^*P* < 0.001.

**FIGURE 4:**
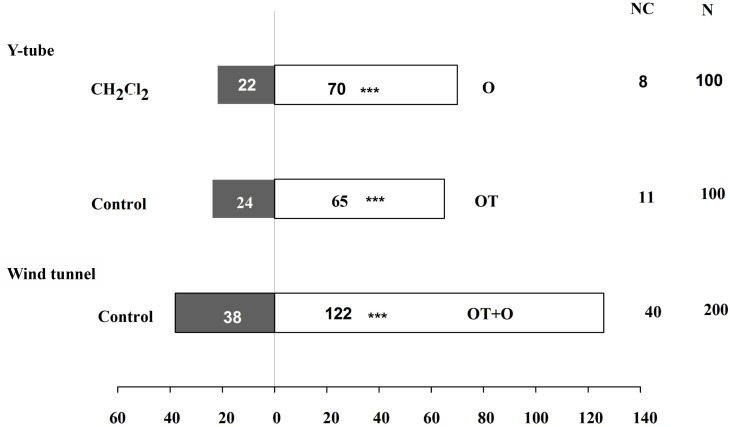
Flight behavior of aphid endoparasitoid, *Aphidius gifuensis* on β-ocimene-treated and control Chinese cabbages. O, purified β-ocimene; OT, β-ocimene treated Chinese cabbage; OT+O, β-ocimene treated Chinese cabbage with purified β-ocimene. Different letters over the bars designate a significant difference: ^∗^*P* < 0.05, ^∗∗^*P* < 0.01, ^∗∗∗^*P* < 0.001.

In addition to our evaluation of the aphid feeding behavior, we also performed two-choice tests to assess the host preference and location behavior of winged aphids and the endoparasitoid *A. gifuensis* in response to β-ocimene-treated and control plants. The number of winged aphids that settled on β-ocimene-treated plants was significantly lower than that on control plants (χ^2^ = 9.981, *P* = 0.002; **Figure 3**). Furthermore, both of the Y-tube olfactometer and wind tunnel bioassays showed that female *A. gifuensis* preferred β-ocimene treated plants over control plants (landing: χ^2^ = 47.220, *P* < 0.001; Preference: χ^2^ = 18.888, *P* < 0.001; **Figure 4**). Moreover, purified β-ocimene proved to be highly attractive to *A. gifuensis* (χ^2^ = 15.506, *P* < 0.001; **Figure 4**).

### β-Ocimene Induced the Expression of SA and JA Marker Genes

**FIGURE 5:**
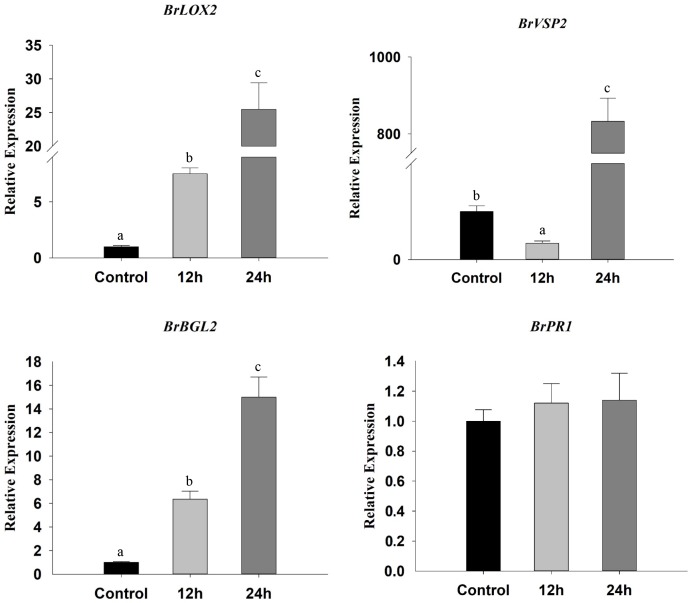
Relative expression levels of SA marker (*BrBGL2* and *BrPR1*) and JA marker (*BrLOX2* and *BrVSP2*) genes in β-ocimene-treated and control Chinese cabbages. OT, β-ocimene treated Chinese cabbage. Different letters over the bars designate a significant difference at *P* < 0.05.

To assess plant defense responses, we not only monitored the expression SA and JA marker genes but also analyzed glucosinolate contents. We found that the expression of JA marker genes *BrLOX2* and *BrVSP2* increased significantly after exposure to β-ocimene, whereas in contrast *BrVSP2* was initially down-regulated (*BrLOX2*: *F* = 125.040, *P* < 0.001; *BrVSP2*: *F* = 562.701, *P* < 0.001) (**Figure 5**). Exposure to β-ocimene also increased the expression of the SA marker gene: *BrBGL2*, whereas it had no detectable influence on the JA marker gene *BrPR1* (*BrBGL2*: *F* = 93.352, *P* < 0.001; *BrPR1*: *F* = 0.905, *P* = 0.480) (**Figure 5**).

### β-Ocimene Triggered the Accumulation of Glucosinolates

**FIGURE 6:**
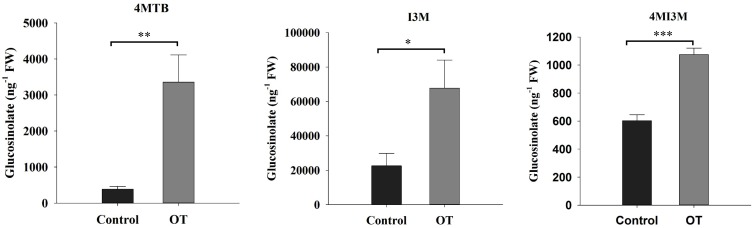
The concentration of glucosinolates in β-ocimene-treated and control Chinese cabbages. OT, β-ocimene treated Chinese cabbage. Different letters over the bars designate a significant difference: ^∗^*P* < 0.05, ^∗∗^*P* < 0.01, ^∗∗∗^*P* < 0.001.

In addition to the JA and SA marker gene expression analysis, we also analyzed the content of glucosinolates at 24 h after exposure to β-ocimene. We found that β-ocimene significantly increased the concentrations of 4MI3M (4-methoxyindol-3-ylmethyl glucosinolate), 4MTB (4-methylsulfinylbutyl glucosinolate), and 1MI3M (1-methoxyindol-3-ylmethyl glucosinolate) in leaves (4MI3M: *t* = -7.491, *P* < 0.001; 4MTB: *t* = -3.899, *P* = 0.002; I3M: *t* = -2.544, *P* = 0.022; **Figure 6**).

### *M. persicae* Adjusted Its Detoxification System to Counter the Defensive Responses of Plants

**FIGURE 7:**
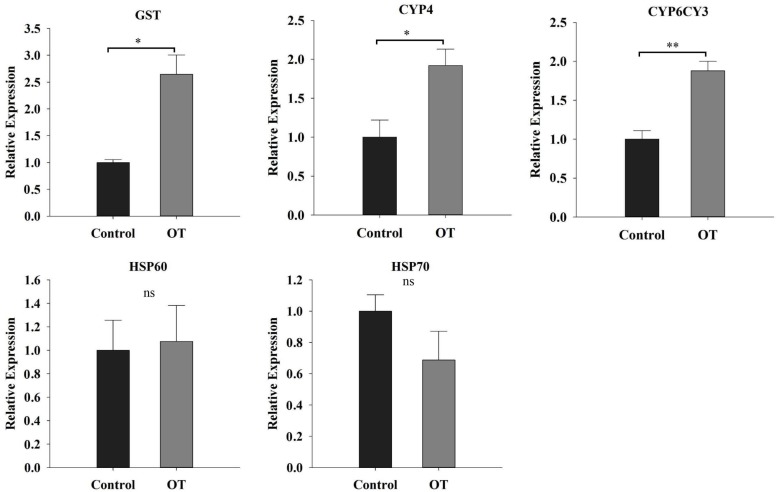
The detoxify response of *M. persicae* reared on β-ocimene-treated and control Chinese cabbages. OT, β-ocimene treated Chinese cabbage. Different letters over the bars designate a significant difference: ^∗^*P* < 0.05, ^∗∗^*P* < 0.01, ^∗∗∗^*P* < 0.001.

Apart from assessing the defense responses of Chinese cabbage, we also investigated detoxification activity in *M. persicae* in response to the β-ocimene induced defense responses in Chinese cabbage (**Figure 7**). We found that when aphids fed on the β-ocimene-treated plants, the expressions of *CYP6CY3*, *CYP4*, and *GST* increased significantly compared with those in aphids that had fed on control plants (*CYP6CY3*: *t* = -7.553, *P* = 0.002; *CYP4*: *t* = -4.212, *P* = 0.014; *GST*: *t* = -4.527, *P* = 0.011). In contrast, feeding on the β-ocimene-treated plants had no detectable influence on the gene expressions of the chaperone and stress responsive proteins *HSP70* and *HSP60* (*HSP70*: *t* = 1.703, *P* = 0.231; *HSP60*: *t* = -0.248, *P* = 0.816).

## Discussion

Hitherto, plant volatiles have been identified as efficient signaling molecules that mediate direct and indirect plant defense responses against herbivores and pathogens (Hare, 2011; Mithofer and Boland, 2012). In the present study, we clearly demonstrated that in Chinese cabbage the volatile β-ocimene induced both direct and indirect defense responses against *M. persicae*. Furthermore, we also explored how *M. persicae* modifies its feeding behavior and detoxification system to cope with the plant defense response.

Initially, we assessed the performance of *M. persicae* on β-ocimene treated plants and the feeding and orientation behavior of aphids. We found that the adult weight and fecundity of *M. persicae* feeding on β-ocimene treated plants were significantly lower than those of aphids that fed on control plants. Moreover, winged aphids showed a preference for landing on the control plants. These results are consistent with observation on *Macrosiphum euphorbiae*, for which it was found that adult weight and fecundity and aphid settlement on tomatoes exposed to E-β-ocimene emitted from transgenic tobacco plants were lower than those of aphid exposed to the control tomatoes (Cascone et al., 2015). Furthermore, exposure to methyl salicylate was found to significantly decrease the population growth of the bird cherry-oat aphid *Rhopalosiphum padi* (Ninkovic et al., 2015). Similarly, exposure to β-ocimene, (E)-2-hexenal, and (Z)-3-hexenal enhanced the resistance of *Arabidopsis* against *Botrytis cinerea* (Kishimoto et al., 2005, 2006, 2008). We found that exposure of *M. persicae* to β-ocimene also impaired their feeding behavior through shortening the total feeding period and phloem ingestion and increasing the frequency of stylet puncture. Consistent with these observation, it has previously been shown that treatment with the insect-produced volatile *E*,*S*-conophthorin significantly reduced the feeding mass of *Solidago altissima* (Helms et al., 2017). Furthermore, exogenous application of chemical deterrents and exposure of nitric oxide also exhibited an impaired feeding behaviors like delayed or failed to reach phloem vessels (Dancewicz et al., 2008; Gabryś et al., 2015; Woźniak et al., 2017).

The aforementioned findings indicate that β-ocimene might induce defense responses in Chinese cabbage, thereby reducing its suitability as a host plant for *M. persicae*. Therefore, we analyzed the expression of genes associated with the JA and SA pathways. We found that defensive responses in Chinese cabbage were activated by β-ocimene treatment, which is consistent with the findings of previous studies on *Arabidopsis* and lima bean (Arimura et al., 2000; Zhang et al., 2012). In these plants, defense-related phytohormone marker genes such as *PR-2*, *PR-3*, *LOX*, and *VSP*, were highly up-regulated after the treatment with β-ocimene (Arimura et al., 2000; Zhang et al., 2012). Similarly, exposure to green leaf volatiles has been shown to activate MAP kinases in *Lolium temulentum* (Dombrowski and Martin, 2014). Gene expression analysis has also revealed that (E)-2-hexenal, (Z)-3-hexenal, (Z)-3-hexenol, and allo-ocimene all activated defense-related genes in *Arabidopsis thaliana* and enhanced its resistant against *B. cinerea* (Kishimoto et al., 2005). Furthermore, bacterial volatiles have been identified to induce systemic plant defenses against many pathogenic bacteria in numerous plant and bacterial species (Pieterse et al., 2013; Sharifi and Ryu, 2016; Tahir et al., 2017). Surprisingly, Arimura et al. (2000) found that only the plant volatiles released by *Tetranychus urticae* infested plants could activate defense response in healthy lima bean, whereas the plant volatiles emitted from artificially wounded leaves did not. Follow-up studies revealed that three terpenoids: E-β-ocimene, (E)-4,8-dimethyl-1,3,7-nonatriene (DMNT), and TMTT were found to be responsible for activation of plant defense response (Arimura et al., 2000). In tea plant *Camellia sinensis* L. and cucumber plants *Cucumis sativus* L., the defense-related gene *LOX* was shown to be induced by treatment with (Z)-3-hexenol, 3-pentanol, and 2-butanone, respectively (Song and Ryu, 2013; Xin et al., 2015). More interestingly, the insect-produced volatile *E*,*S*-conophthorin has been shown to elicit the accumulation of JA and SA in *S. altissima* (Helms et al., 2017). In addition, the accumulation of SA in tomato is adversely associated with aphid feeding performance and preferences (Avila et al., 2012). Thus, all of these results revealed that specific plant volatiles are able to prime plant defense responses to fend off the attackers.

In addition to defense genes, we also examined the glucosinolate contents in Chinese cabbage, which have been identified as the key defense metabolites in *Brassica* species (Kim and Jander, 2007; Hopkins et al., 2009; Kliebenstein, 2012). We found that the content of 4MI3M, glucoerucin and glucobrassicin were all significantly increased in plants exposed to β-ocimene. In previous studies, aphid feeding has also been demonstrated to induce the accumulation of 4MI3M, and the indole glucosinolate I3M has been identified as an aphid deterrent in *Arabidopsis* (*M. persicae*), *Brassica oleracea* (*Brevicoryne brassicae*) and Chinese cabbage (*M. persicae*) (Kim and Jander, 2007; Hol et al., 2013; Cao et al., 2016). Furthermore, MeJA treatments have been shown to increase the concentrations of I3M, 4-methylsulphinylbutyl glucosinolate and 1-methoxyindol-3-ylmethyl glucosinolate in cauliflower curds, whereas SA, but not MeJA, was found to induce the accumulation of 4MI3M and 4-hydroxyindol-3-ylmethyl glucosinolate in Chinese cabbage (Ku et al., 2013; Cao et al., 2016). In *B. oleracea*, higher levels of five glucosinolates negatively influenced the population doubling time of *B. brassicae* (Hol et al., 2013). Similarly, in the present study, we demonstrated that exposure to β-ocimene negatively affected the feeding behavior of *M. persicae*, which was revealed by our EPG data. We found that when feeding on β-ocimene-treated Chinese cabbage, *M. persicae* exhibited a longer pre-feeding period and spent less time ingesting phloem sap, which indicated that β-ocimene-treated plants had acquired a certain degree of resistance. In contrast, in *Arabidopsis*, both of aliphatic and indole glucosinolates were observed to be beneficial to *B. brassicae*, while negatively influencing the performance of the predacious hoverfly *Episyrphus balteatus* (Kos et al., 2012). In addition, Cao et al. (2016) have hypothesized that aphid feeding induced higher nutrient accumulation, which might weaken the inhibitory effect of glucosinolates on aphid performance. Thus, all of these results indicated that the accumulation of glucosinolates might contribute to be the deterrent effect and restrict the performance of *M. persicae*, as observed in the present study. Nevertheless, in addition to glucosinolates, it is probable that *B. oleracea* and Chinese cabbage contain other defense-related metabolites. For example, in tomato, (Z)-3-hexenylvicianoside (HexVic) has been identified as a defense metabolite against *Spodoptera litura* (Sugimoto et al., 2014). Furthermore, exposure to (Z)-3-hexenol has been shown to significantly induce the accumulation of HexVic in plants (Sugimoto et al., 2014). The findings of these studies thus indicated that exposure to β-ocimene might reduce the fitness of *M. persicae* through the accumulation of defense-related metabolites in Chinese cabbage.

Apart from the direct defense response, we also assessed the preference of *A. gifuensis* wasps when given choice between β-ocimene-treated plants and control plants. We found that *A. gifuensis* showed a preference for β-ocimene-treated plants compared with the healthy control plants. Consistent with this observation, it has previously been shown that tomato exposed to β-ocimene emitted from transgenic tobacco plants proved to be more attractive to *A. ervi* than control plants, which possibly resulted from the higher emission of volatiles from tomatoes exposed to the β-ocimene emitted from transgenic tobacco plants (Cascone et al., 2015). Similarly, E-β-ocimene emitted from transgenic tobacco plants has also been found to increase the emission of volatiles in lima bean (Arimura et al., 2012). Furthermore, exposure to (Z)-3-hexenol was found to elicit the release of β-ocimene and linalool and enhance the attractiveness of tea geometrid *Ectropis obliqua*-infested tea plants to *Apanteles* sp., the main parasitoid wasp of this moth (Xin et al., 2015). Similarly, exposure to the plant volatiles emitted by artificially damaged *Arabidopsis* plants significantly increased the attractiveness of healthy *Arabidopsis* plants to *Cotesia glomerata*, which is a parasitic wasp of the cabbage white butterfly *Pieris rapae* (Shiojiri et al., 2012). In this regard, the results of our Y-tube bioassays also confirmed the capacity of pure β-ocimene to attract natural enemies of insect pests. Likewise, the tephritid parasitoid *Psyttalia concolor* has been found to be strongly attracted to β-ocimene released from olives in response to infestation with *Bactrocera oleae* (Giunti et al., 2016a,b, 2018). All of these results indicated that certain specific volatile compounds can be considered as important players in the plant–plant and plant–insect interactions that occur during biotic and abiotic stress.

In response to β-ocimene-induced defense responses, *M. persicae* also adjusted and optimized its feeding behavior and detoxification enzyme system to reduce the detrimental side effects (Schuler and Berenbaum, 2013). In *M. persicae*, *CYP6CY3* has been identified as being involved in the resistance to nicotine and neonicotinoids (Silva et al., 2012; Bass et al., 2013; Peng et al., 2016a), and Bass et al. (2013) found that overexpression of *CYP6CY3* led to a significantly higher resistance to nicotine and neonicotinoids. Furthermore, in their subsequent work, these authors revealed that when *CYP6CY3* is heterologously expressed in Sf9 insect cells, it can metabolize nicotine and neonicotinoids to less toxic products (Bass et al., 2013). Similar to *CYP6CY3*, *CYP4* is another key detoxification CYP family in insects (Silva et al., 2012). In an insecticide-resistant morph of the Asian citrus psyllid *Diaphorina citri* Kuwayama, the gene expressions of CYP4 family members were significantly higher than those in the susceptible morph (Chen et al., 2018). In addition to the CytP450-dependent detoxification system, we also found that feeding on β-ocimene-treated plants induced the expression of GST in aphids, which has been identified as being involved in the adaptation to plant defensive metabolites in various insect species, including *M. persicae* (Francis et al., 2005). Francis et al. (2005) found a significant induction of GST activities in *M. persicae* reared on Brassicaceae plants and artificial diets containing glucosinolates. Furthermore, our EPG data also revealed that *M. persicae* could alter their feeding behavior, such as shortening feeding time to reduce the ingestion of glucosinolates. Moreover, we observed that *M. persicae* also spent more time in selecting a feeding area. All of these results indicated that *M. persicae* can modify its feeding behavior and detoxification machinery to counter the defensive responses of plants.

In terms of pest control, the key defense-related volatile components can potentially be used as a perfect complement to the application of natural enemies. Firstly, these key volatile components prime the defense responses of target crops to increase their resistance to the pest. Secondly, they not only serve as deterrents of pest but also as attractants of the natural enemies of these pests. For example, in an open field experiment, it was shown that the application of plant volatiles 3-pentanol and 2-butanone significantly increased the number of ladybird beetles, *Coccinella septempunctata* (Song and Ryu, 2013). Furthermore, release of these volatiles in heavily infested areas or in areas at the initial stage of infestation can reduce the search time of these natural enemies to increase their biological efficiency. Thus, combining the application of key volatiles with the release of natural enemies could potentially contribute an effect integrated pest management strategy.

In a summary, on the basis of our findings, we can conclude that exposure to β-ocimene induces defense-related genes and downstream metabolites in Chinese cabbage to restrict the fitness of *M. persicae* and influence the preference of *A. gifuensis*. Our current work has revealed that the key components of plant volatile can trigger the biochemical and molecular responses in plants that enhance their resistance to both biotic and abiotic stresses. In the future, we should seek to identify further principal volatiles compounds and evaluate the eco-agricultural application of these compounds in both the field and greenhouse.

## Author Contributions

Z-WK and T-XL designed the research. Z-WK, F-HL, and Z-FZ performed the research. Z-WK and H-GT analyzed the data. Z-WK and H-GT wrote the manuscript. Z-WK and T-XL edited and revised the manuscript.

## Conflict of Interest Statement

The authors declare that the research was conducted in the absence of any commercial or financial relationships that could be construed as a potential conflict of interest.
